# Organic Food Consumption and Perception among Polish Mothers of Children under 6 Years Old

**DOI:** 10.3390/ijerph192215196

**Published:** 2022-11-17

**Authors:** Karolina Woś, Hubert Dobrowolski, Danuta Gajewska, Ewa Rembiałkowska

**Affiliations:** 1 Department of Functional and Organic Food, Institute of Human Nutrition Sciences, Warsaw University of Life Sciences (SGGW), 02-776 Warsaw, Poland; 2 Department of Dietetics, Institute of Human Nutrition Sciences, Warsaw University of Life Sciences (SGGW), 02-776 Warsaw, Poland

**Keywords:** organic food, organic consumption in Poland, sustainable consumers, organic diet, women’s diet

## Abstract

Pro-environmental attitudes, including organic food consumption, can reduce negative impact on the environment. The consumption of organic food in Poland is rather low, but the ecological awareness of Poles is steadily increasing. The aim of the study was to assess the frequency and factors influencing the consumption of organic products and to analyze the perception of such food by mothers of children under 6 years of age (*n* = 667). The survey was conducted between March 2020 and May 2021 in three voivodships in Eastern Poland. The results of the survey indicate that the majority of respondents are occasional consumers of organic food (51%). The most commonly consumed organic products are eggs, vegetables and fruits, whereas the least consumed are alcoholic beverages, coffees and ready-to-eat meals. The responders’ main characteristics of organic food are as follows: no genetic modification/GMO-free, no synthetic additives and having organic certification. Statistically significant correlations were found between the frequency of organic food consumption and education, financial situation as well as familiarity with the logo of the organic certificate and verifying that it is present on the packaging. The most common reasons for consuming organic food were health issues, while the high price was declared as the main barrier.

## 1. Introduction

Climate change and food safety and quality are challenges facing modern society and future generations. The response to these challenges is the need for sustainable consumer behaviors, including informed and sustainable food consumption. The environmental awareness of European consumers has been growing over the last decade. They declare a growing interest in sustainable consumption and pro-environmental characteristics of products, which increases the willingness to pay for organic products [1,2,3,4]. According to the meta-analysis by Li and Kallas (2021), consumers can pay 29.5% more on average for sustainable product features in comparison with the regular conventional products [5].

Organic food production is sustainable, and organic foods are rich in health-promoting substances while containing fewer harmful substances [6,7,8,9,10]. A well-balanced diet based on organic food reduces the risk of certain diseases, including obesity and cancer. Regular consumers of this type of food have the greatest health benefits [7,11,12,13]. Moreover, the composition and health-promoting properties of organic food make it particularly important in nutrition during pregnancy [11] and infancy [14]. The pre- and postnatal development periods are characterized by a very high sensitivity to the composition of the food consumed. According to metabolic programming theory, environmental and nutritional factors acting during this critical time have a significant impact on health in later life [15,16,17,18]. Therefore, the most frequent group of organic food consumers are families with young children, where women are predominantly responsible for composing and preparing meals for the whole family [19], but their nutritional knowledge is not always sufficient [20,21,22]. However, experts and producers claim that the interest in these products is growing, and recent studies show that there are significant relationships between the purchase (and consumption) of organic products and perceptions of organic food (healthiness, trustworthiness, quality, control system, authenticity, safety) as well as increasing pro-environmental consumer attitudes [23].

According to the Report by the BIO Coalition in cooperation with Nielsen IQ (2021), the value of the organic food market in Poland is estimated at PLN 1.36 billion, representing 0.5% of the food market. Over 32% of consumers declare buying organic food “at least once a week” and “at least once a month”, while 48% declare never buying this type of food. Declared expenses on organic food were on average 194 PLN/household per year in Poland in 2019 (just over EUR 45 as of 2019) [24]. For comparison, according to The Research Institute of Organic Agriculture (FiBL) data for 2020 expenses for organic food consumption per year in Poland was EUR 8/person, compared to EUR 19 in the Czech Republic, EUR 180 in Germany and as much as EUR 384/person/year in Denmark [25]. In Poland, the consumption of organic food is not popular, and organic products are not widely available. The typical consumer of organic food is a woman, aged 36–45 years, with higher education, good or very good financial status and living in a city [19,24].

Despite the intensive development of organic food processing and trade in Poland, information on the organic food market is difficult to access due to the lack of a coherent system for collecting data on production, distribution and consumption of organic products. The data collection process is not standardized, and it is often impossible to compare these data [26]. 

There are only few studies evaluating the consumption of organic food by Poles [19,24,27,28,29,30]. The IMAS research (2017) conducted on a nationwide sample (518 people) indicates which of the 17 listed product groups are purchased most frequently by Polish consumers. It was also investigated that consumers typically spend between PLN 20 and PLN 100 at a time when shopping for organic products. According to this report, 24% of Poles buy at least once a week, 29% at least once a month, 15% at least once every three months, 11% at least once a year, 12% less than once a year and 9% never [19].

A study by Hermaniuk (2018) included 377 randomly selected Polish citizens. It showed that 13.4% consume organic food daily, 23.9% several times a week, 25% several times a month, 28.7% several times a year and 9.1% never. It was also shown that consumers would like to buy organic fruit and vegetables, meat and cold cuts and bread more often than other products of the 13 listed organic product groups [27]. 

According to a study by Jarczok-Guzy (2019) conducted on 1159 inhabitants of the Silesian voivodeship (Southern Poland), consumers most often bought meat and meat products, cereal products and vegetables and fruit. Overall, 30% of the respondents bought ecofood once a week, 38% once a month, 17% every 3 months on average, 8% every 6 months, 4% once a year and 3% less than once a year. None of the surveyed group declared that they never buy organic food [28].

In a study by Kułyk and Michałowska (2019), 500 randomly selected Polish residents were surveyed. Of this number, 198 people, i.e., approximately 40%, had never bought organic food. Of the remaining 302 people, 55% bought once a week, 25.8% bought 2–3 times a week, 14.9% bought once a month and 4.3% bought twice a month. This way of reporting makes the data incomparable with the results of the above-mentioned authors, except for the answer “I never buy organic food” [29]. 

In a paper of Kułyk and Dubicki (2019), 211 randomly selected people from the Lubuskie voivodship (Western Poland) were surveyed. Here, it was also shown that the most frequently purchased products were eggs, vegetables and fruit. As far as the frequency of buying organic food is concerned, the authors provide very limited data. The majority of respondents (66.4%) declared that they buy organic food at least once a month. The group of consumers who never buy organic products accounted for 12.3% [30]. 

According to a report by the BIO Coalition (2021), just over 32% of consumers declared that they buy organic food “at least once a week” and “at least once a month” and can thus be counted as regular consumers of organic food. In contrast, 20% reach for organic food occasionally and 48% say they never buy organic food. The highest share of consumers who declared that they buy organic food was recorded in the Śląskie, Mazowieckie, Wielkopolskie and Łódzkie voivodships. These are the areas of Central, Southern and Western Poland. According to this report, the most frequently purchased products are fruit, vegetables, eggs and bread [24].

Based on the described literature, it can be concluded that there is little research evaluating the frequency of organic food consumption by the Polish population.

Furthermore, there is no uniform system for assessing the frequency of consumption of organic food and each group of authors uses its own separate system. The data obtained are highly divergent from one study to the next, e.g., the percentage of respondents declaring that they never buy organic food varies from 0 to 48% in different studies. This is probably due to the inconsistent data acquisition methodology. The groups of respondents surveyed differed in size, the way the group was selected and the area of Poland they inhabited. Some of the surveys were nationwide, while others were local, concerning one voivodship. All this makes it impossible at present to draw consistent conclusions about the frequency of buying/consuming organic food in relation to the statistical resident of Poland.

In addition, it should be noted that none of the works cited above analyzed the consumption of individual product groups in such detail as in the presented study, nor did they survey so many women. Finally, previous studies included respondents who were not verified as to their knowledge of organic production principles as in our study. In the research to date, respondents declared eating organic food, but it is not clear whether they actually knew what organic food was. The often declared buying of organic food in open-air markets and bazaars may indicate buying regular conventional food under the organic signboard. This is probably one of the reasons why the data obtained by the cited authors differ so much. It should be emphasized that no paper was found in databases on the frequency of consumption of organic food in Eastern Poland.

Therefore, the authors of this study felt that a detailed study of women living in the eastern provinces of Poland with young children was justified. The rationale for choosing this group of respondents is the strong influence of food composition on the development and health of young children, as described above. It was decided to include only those who know what organic food is in order to obtain reliable data on the frequency of consumption of organic food.

The study is therefore an innovative contribution to knowledge on the consumption of organic food in Poland, with particular emphasis on the eastern region, which has not been studied in this respect so far. The obtained results will allow us to extend the real knowledge about the consumption of organic food and the factors influencing it, which, in turn, will allow us to build further strategies for increasing the consumption of organic food in Poland.

The aim of the study was to assess the frequency of consumption of selected groups of organic products by mothers of children under 6 years of age living in the provinces of Eastern Poland. Selected factors influencing the consumption of organic food were also evaluated, as well as the perception of such food by the women surveyed.

The following research hypotheses were set:

**Hypothesis** **1.** *Most of the female respondents consume organic food occasionally*.

**Hypothesis** **2.** *Socio-economic factors (age, education, financial situation, residence, number of people and number of children under 6 in the household) significantly influence the consumption of organic food*.

**Hypothesis** **3.** *The most common reason for consuming organic food is health issues, and the most common barrier to consuming organic food is its high price*.

## 2. Materials and Methods

The survey was conducted among the beneficiaries of the “BIO for Mother and Child” research and education project implemented by Warsaw University of Life Sciences between 1 September 2019 and 30 April 2022. Its main objective was to encourage the consumption of organic food in Eastern Poland by providing nutritional education in this area to the largest possible group of young mothers (pregnant women and mothers of children up to 6 years old). The research and all procedures were approved by the local Ethics and Scientific Research on Humans Commission at the Institute of Human Nutrition Sciences of the Warsaw University of Life Sciences (approval number: 08/2020; 6 July 2020). All participants consented to participate in the study.

### 2.1. Study Group

The survey was conducted in the period from March 2020 to May 2021 on the target group of the “BIO for Mother and Child” project, among adult pregnant women (over 18 years of age) and/or mothers of children up to the age of 6 residing in the following voivodeships of Eastern Poland: Mazowieckie, Lubelskie, Podlaskie, Warmińsko-Mazurskie and Podkarpackie. 

A total of 1142 completed surveys were collected from this group. Respondents with vocational and primary education were excluded from the study due to very low numbers (7 people), and questionnaires of respondents who indicated an incorrect definition of organic food (468 people) were also excluded. Women who did not indicate the true definition of organic production were excluded in order to avoid a situation where a woman claims to have organic food in her diet, but in fact it is not organic at all. Respondents who were unable to identify organic food could not guarantee that they actually consumed this food. Finally, 667 questionnaires were analyzed in this study. Figure S1, a flow chart of the project in the Supplementary Materials, shows the selection scheme of the respondents for this study.

### 2.2. Research Tools

The research tool was a questionnaire conducted in two ways:

1. Printed version distributed in selected establishments (the PAPI method) (89% of respondents);
2. Online version available on the project website (the CAWI method) (11% of respondents).

The questions in the survey allowed us to assess the frequency of consumption of organic food and the basic knowledge and beliefs about organic food. The questionnaire included the following:

- Organic food section questions included the following: understanding the term “organic food”, identifying the characteristics of organic food (possibility to choose up to 4 characteristics from those listed), frequency of consumption of organic food according to the 24 groups measured on a 6-point scale from “several times a day” to “never”, identifying whether respondents make sure that the logo of certified organic food is on the packaging when buying organic products, selecting the official label for certified organic food within the European Union (from 8 sample labels), selecting the 3 most important reasons for eating organic food, selecting the 3 most important barriers to eating organic food and how much more the respondent is willing to pay for certified organic food compared to regular conventional food; additionally, this section contains questions asking for views on (a) the certainty that organic food has been produced according to organic production rules only for certified food and (b) the possibility to use the organic food logo on packaging only after obtaining permission from the relevant authorities.
- Demographic characteristics section included questions on the following: age, place of residence, voivodeship of residence, education, number of people in household, number of young children (under 6) in household, pregnant at time of questionnaire completion, civil status, education and financial situation.

### 2.3. Transformation of Data on Organic Food Consumption

The data from the question on the frequency of consumption of the 24 selected organic product groups were “question-scales” with increasing frequency of consumption from “never” to “several times a day”. The six original food intake frequency categories were transformed into semi-quantitative data that logically reflect the increasing intensity of the trait using a transformation into numbers and expressing food intake frequency as multiples/day, as shown in Table 1.

This way of compiling the results using daily frequency indices expressed as multiples/day is a validated methodology for transforming dietary data, which was derived from a questionnaire to survey dietary views and habits for people aged 16 to 65 years developed by the Behavioral Determinants of Nutrition Team of the Committee on the Science of Human Nutrition of the Polish Academy of Sciences [31]. 

The 24 original organic product groups were also re-structured, categorizing them into a collective 17 food groups, including bread, cereal products, milk, dairy products, eggs, vegetables, fruit, vegetable and fruit products, processed meats, meat, fish, legumes, ready meals, confectionery, honey, coffee and tea and alcoholic beverages.

The consumption of each of the 17 product groups corresponded to the daily frequency (times/day) allocated according to the respondent’s declared frequency of consumption according to Table 1. For the collective product groups indicated above, e.g., processed cereals, the daily frequency was calculated based on the average of all the product groups comprising the collective group. 

The daily consumption frequencies of the 17 product groups were then summed up for each respondent and, based on the result, the respondents were categorized in terms of their level of consumption of the selected organic products according to a six-degree scale ranging from “no consumption of organic products” to “very high level of consumption of organic products” as shown in Table 2. A higher sum of daily frequencies corresponds to a higher level of consumption of the organic product groups analyzed. The increasing level of consumption is also linked to the increasing impact on the quality of the respondent’s diet and the market for organic food. Based on the assumed consumption levels of the analyzed product groups, 3 categories of female organic food consumers were also determined: “regular eco-con”, “occasional eco-con” or “non eco-con” (Table 2). Regular eco-con included all respondents who consume organic products at least once a week, and their level of consumption of selected eco-products was medium, high or very high. The occasional eco-con group included respondents who declared consuming the analyzed products less frequently than once a week, with a low or very low level of consumption. Only respondents who declared that they do not consume any of the analyzed organic food groups and characterized by a complete lack of consumption of the analyzed organic products were included in the non-eco-con.

### 2.4. Statistical Analyses

The data obtained from the survey were transferred to an Excel spreadsheet and then processed by being structured, checked and validated. The data were coded and statistically analyzed using SPSS v. 28.0 software (IBM corp., Armonk, NY, USA). To verify the normality of distribution, the Shapiro–Wilk test was used. The Chi-squared test was used to compare the groups. To compare obtained results on the amount of organic food consumed between different groups, the Kruskal–Wallis test was applied. The study’s defined significance level was set to α = 0.05.

## 3. Results

This study analyzed surveys in which female respondents ticked the correct definition of organic food as: “food produced without the use of synthetic fertilizers and plant protection products and without genetic modification”, which accounted for 59% of the surveys. This approach resulted in more reliable data—responses from female respondents unable to correctly define organic food were not analyzed. Other respondents defined organic food as “food produced on small family farms run using natural methods” (23%), “food produced in a clean, unpolluted agricultural environment” (16%) or “food purchased at a market, bazaar or on the street” (2%).

Table 3 shows the structure of the study group. Participants in the study are predominantly women with a high level of education, good and very good material status, living in small towns (10,000–100,000 inhabitants), married, with one child under 6 years and with four family members in the household.

Table 4 shows the results on the declared consumption of the 24 organic product groups analyzed in the last 3 months. For 21 of the 24 organic product groups, the majority of female respondents indicated consumption with a frequency of “never”. The least frequent organic products consumed are alcoholic beverages, ready meals and coffees. The highest proportion of regular eco-consumers was recorded for the consumption of organic eggs, with a total of 70.8% of female respondents declaring to consume them at least once a week. Vegetables (58.9% of respondents) and fruit (49.3%) also stand out from the organic products analyzed, as do processed vegetables (38.5%), honey (38.4%), processed fruit (35.8%) and cured meats (31.6%). Among the product groups analyzed, female respondents are less likely to consume products such as organic bread (26.0%), white meat (26.8%), fermented dairy drinks (25.7%), buckwheat groats, oatmeal and whole grain pasta (23.4%). Regular consumption of the other product groups was reported by a small proportion of female respondents.

Based on the data in Table 4, the consumption of organic food of female respondents was determined by categorizing them according to their level of consumption and eco-consumer category. The research showed (Table 5) that the largest group is made up of female respondents with an average consumption of organic food (37.9%), but there is also a large proportion of women with very low and low consumption. The median intake for the entire group of female respondents is 1.9 times/day, which, according to the accepted categorization, corresponds to low intake. According to the categorization adopted, occasional eco-consumers account for the largest share with 51.3% (Table 5).

Table 6 shows the relationship between selected socio-economic factors and consumption of organic food. A statistically significant relationship was detected between organic food consumption and factors such as educational level (*p* = 0.041) and financial situation (*p* = 0.038). No statistically significant correlations were detected between the other socio-economic factors analyzed (*p* > 0.05). A trend towards slightly higher consumption was found in the following groups: younger mothers—under 35 years of age, those with higher education and those with a good or very good financial situation. Higher consumption was noted as a trend for residents of rural areas and towns with up to 10,000 inhabitants and among residents of the Podlaskie voivodeship. With regard to the household, a trend towards slightly higher consumption was detected in larger families with at least five people living at home and with more than one child under 6 years of age, but also in families where the mother is raising a child alone (single/divorced/widowed).

Table 7 shows the relationships between groups in knowledge of selected consumer issues related to organic food consumption. Of the factors analyzed, two were found to be significant: checking for the presence of the certificate on the packaging when buying organic products (*p* < 0.001) and knowledge of the organic certificate (*p* = 0.004). It was noted that the regular eco-con group had the highest proportion of people who check for the presence of a certificate and correctly indicate the organic food logo, while the lowest proportions of these people were characteristic of the non-eco-con group. In total, 27% of female respondents do not check the presence of the certificate on the packaging and 15% cannot correctly identify the organic food logo. Furthermore, a total of 43% of female respondents do not confirm that only with certified products can one be sure of compliance with organic production rules, and 22% do not confirm that it is necessary to obtain permission from the relevant authorities to use the organic food logo on the product packaging.

Table 8 shows the most frequently indicated characteristics of organic food and the proportion of respondents who indicated them. Among a possible maximum of four characteristics, the most frequently indicated by respondents were no genetic modification/no GMOs, no synthetic additives and having organic certification. Environmental friendliness and the content of beneficial nutrients were indicated by an average of one in five/one in six respondents, indicating a rather low state of knowledge among the women surveyed about the actual qualities of organic food.

Table 9 shows the reasons for consuming organic food by the female respondents surveyed. The most common reasons for choosing organic food are its health properties and the absence of artificial additives, with a total of 54.1% of indications.

Table 10 indicates the barriers that most prevent respondents from eating organic food. Among these, the most important are the high price and the unsatisfactory offer of organic food. About one-fourth of the indicated barriers are the preference to buy local products (at the market or marketplace) or to grow them in one’s own cultivation.

**Figure 1:**
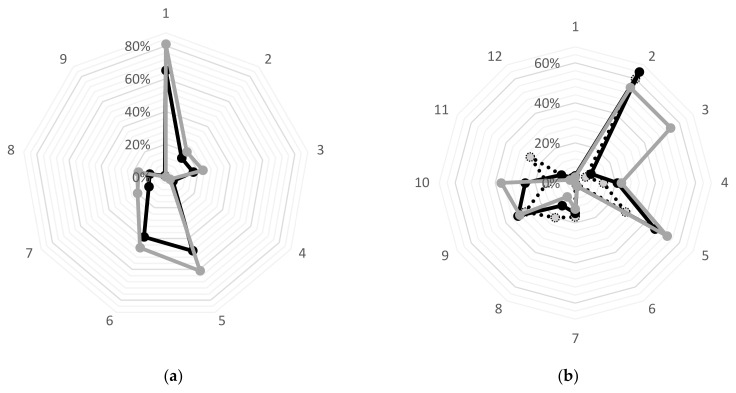
Reasons and barriers to consumption of organic food of respondents. (**a**) The reasons for consuming organic food (% of respondents) are as follows: (1) it is healthier, (2) it is tastier, (3) it contains more nutrients (vitamins, minerals, etc.), (4) it helps to treat diseases/allergies, (5) it does not contain artificial additives (preservatives, flavors, colors), (6) it is not genetically modified (without GMO), (7) out of concern for the environment, (8) I want to support organic farming and (9) it is a trendy food. (**b**) The barriers to consumption of organic food (% of female respondents) are as follows: (1) I do not know how to recognize organic food/I do not know how it is labelled, (2) organic food is too expensive/not worth the price, (3) not available/I have not seen it in shops, (4) organic food shops are too far away, (5) there is not enough choice of organic products in shops, (6) organic food is no different from conventional food, (7) I do not know the producers of organic food, (8) I do not trust certificates on products, (9) I prefer to buy products from a local farmer or market, (10) I prefer home-grown food, (11) I have no need for it/it does not matter to me and (12) I am disappointed with the quality/taste. Grey line = regular eco-con (*n* = 290); black line= occasional eco-con (*n* = 342); dashed line with grey bullet = non eco-con (*n* = 35).

Figure 1 shows the reasons and barriers for consuming organic food in terms of frequency of consumption. Figure 1a shows that the reasons for consuming organic food are quite universal for both occasional and regular eco-consumers, as the lines of the graphs line up very similarly. The importance of the analyzed reasons is generally higher for regular eco-con. They were noticeably more likely to specifically indicate two reasons such as that organic food (1) is healthier (81% vs. 65%) and (5) does not contain artificial additives (61% vs. 48%) (Figure 1a). Figure 1b shows that the barriers to organic food consumption vary according to the frequency of consumption, as the lines of the graphs differ. Non-eco-con were most likely to respond that the biggest barriers for them were as follows: (8) I do not trust certificates on products (20% vs. 13% in the occasional group and 8% in regular eco-con) and (11) I do not need it/it does not matter to me (26% vs. 8% and 3%, respectively). Occasional eco-con most frequently indicated that (2) organic food is too expensive/not worth it (64% vs. 60% non-eco-con and 55% regular eco-con). In contrast, the barriers most commonly faced by regular eco-con are as follows: (3) not available/I have not seen it in shops (16% vs. 6% non-eco-con and 9% occasional), (5) there is too little choice of organic products in shops (53% vs. 29% and 46%, respectively) and (10) I prefer home-grown food (37% vs. 14% and 25%, respectively) (Figure 1b).

**Figure 2:**
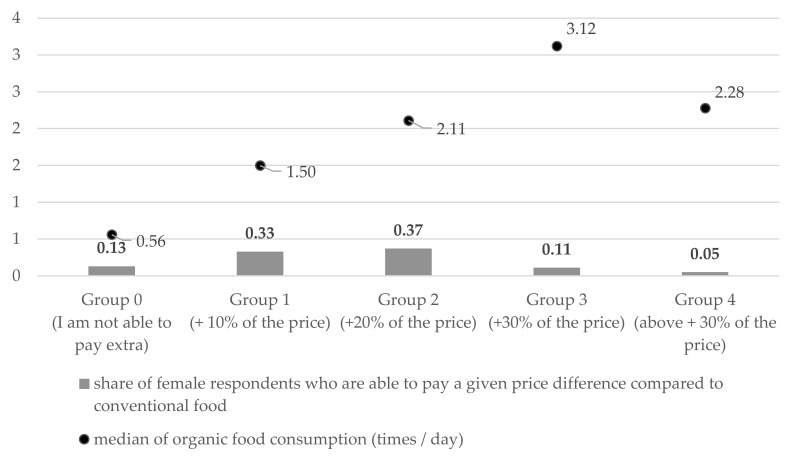
Willingness of the respondents to pay a higher price for certified organic food compared to the price of conventional food in relation to median consumption of organic food (*n* = 667).

Figure 2 shows how much more respondents are willing to pay for a certified organic product compared to the same but conventionally produced food. Most respondents (70%) indicated that they were able to pay around 10–20% more of the price, but as many as 13% were not able to pay more at all. It was also observed that the willingness to pay (WTP) a higher price for organic products increases with median consumption (Figure 2). This trend is confirmed by the detected statistical differences between Group 0 and Group 2 (*p* = 0.005), Group 3 (*p* < 0.001) and Group 4 (*p* = 0.004) and between Group 3 and Group 0 (*p* < 0.001) and Group 1 (*p* = 0.004).

## 4. Discussion

The purpose of our study was to assess the frequency of consumption of organic products by a group of mothers of children under 6. It is one of the few studies describing this issue, and one of the first to highlight in such detail the structure of consumption in relation to a number of socio-economic parameters and consumer attitudes towards organic products in a group of mothers of children under 6 living in the eastern part of Poland.

It should be noted that there are no standards or guidelines that define regular consumption of organic food. According to the criteria given in the paper of Baudry et al. (2019), a low proportion of organic food was considered to be <10%, while a high proportion was considered to be >50% in the diet (expressed as the proportion of organic food in the total diet in g/day) [32]. In polish report IMAS (2017) as “frequent consumers” qualifies those who consume organic food at least once a month [19]. In the Coalition for the BIO Report, “regular consumers” are those who consume at least once a month, while consumers with lower consumption are described as “occasional” [24].

Therefore, the authors of this paper had to adopt their own criteria to define regular consumer of organic food, only partly based on those of other authors described above. It has been assumed that consumption of organic produce at least once a week can be considered regular consumption, while less frequent consumption is occasional. In our opinion, eating organic food once a month is not a basis for considering someone as a regular organic consumer.

In terms of potential impact on consumer health, our understanding of regular consumption is closest to that of Baudry et al. 2019, who consider >50% organic food in the diet to be a large proportion. These authors showed that, comparing to occasional consumption, frequent consumption of organic food was associated with significantly higher plasma concentrations of a number of biomarkers—magnesium, fat-soluble compounds and vitamins (α-carotene, β-carotene, lutein and zeaxanthin) and fatty acids (linoleic acid, palmitic acid, γ -linolenic acid and DHA). Simultaneously, fatty acid desaturase indexes were more favorable with frequent consumption [32].

The results of the presented survey confirm hypothesis 1: Most female respondents consume organic food occasionally (51.3%). It is worth noting that the term “occasional consumption” is defined here as consuming, on average, each of the organic product groups analyzed with a frequency of less than once a week. It is to be presumed that such occasional frequency of consumption has little impact on the organic food market and on the quality of the consumer’s diet. 

At the same time, the female respondents of the present study were characterized by a higher consumption of organic products compared to other Polish studies. Respondents with an average level of consumption (at least once a week) make up the largest proportion of the study group (37.5%), while 5.5% consume eco food more than once a week (Table 5). In comparison, in Hermaniuk’s (2018) study, as many as 62.7% of respondents claimed to consume organic food no more than a few times a month, and only 23.9% claimed to consume it several times a week [27]. According to the Coalition for the BIO Report (2021), around 13% of consumers declared that they buy organic food “at least once a week” [24]. At the same time, the survey also reported a lower proportion of women never consuming organic food (5.2% of respondents), compared to 8% of women not consuming organic food in IMAS study (2017), 9.1% of respondents in Hermaniuk (2018) study or around 40% according to the Coalition for the BIO Report (2021) and the study by Kułyk and Michalowska (2019) [19,24,27,29]. The higher consumption may be due to the characteristics of the study group and the fact that they are women with young children. According to the study conducted by Witek (2017), women who are mothers of young children have more favorable attitudes than men towards the environment and organic products, and concern for the health and safety of the family, especially children, is a strong motivator for purchasing organic products [33]. Tobler et al. (2011) also found that women were more likely to adopt organic food consumption patterns [34].

The presented study analyzed three voivodeships of Eastern Poland. Occasional consumption dominated in each of them, but consumption at least once a week was declared by 39.5% of respondents in the Mazowieckie voivodeship to 52% in the Podlaskie voivodeship. The high consumption in the Podlaskie voivodeship may be due to the fact that, according to the latest Agricultural and Food Quality Inspection report, in 2020, it was second in Poland in terms of the number of organic producers; the Podlaskie voivodeship had 2953 producers, which accounted for 14.6% of the total number of organic producers in Poland; the Mazowieckie voivodeship has 2661 producers, and the Lubelskie voivodeship had 958 [35]. When comparing the level of consumption, it is also worth relating it to Western Poland (Silesian voivodeship), where once-a-month consumption (38%) dominated among 1159 consumers, and once-a-week organic food was consumed by 30% of respondents [28]. Consumption in the provinces we analyzed was therefore more frequent, but it should be noted that this is a survey conducted among mothers of young children, who are a specific group of consumers.

The identified organic product groups consumed most frequently—eggs, vegetables and fruit (and their processed products) (Table 4)—are also indicated in many other studies, and usually the consumption of these products is the highest [19,24,27,30]. 

Research hypothesis 2, which states that socio-economic factors (age, education, financial situation, place of residence, number of people and number of children under 6 in the household) significantly influence the consumption of organic food, was not fully confirmed in this study. In this study, statistically significant relationships were confirmed with regard to educational level and financial situation. However, no relationship was observed between the other socio-economic factors and the consumption of organic food.

Previous research has defined the eco-consumer as one with higher education [19,36]. The result of our study confirms this trend. In the group with higher education, there was a statistically significantly lower percentage of respondents not consuming organic food at all and a higher percentage of regular consumers of such food (*p* = 0.041). The median consumption of organic food was significantly higher among respondents with higher education (*p* = 0.036).

The results indicate that financial situation can have a statistically significant effect on organic food consumption. The median consumption of organic food in the group with the highest financial status is statistically significantly different (*p* = 0.016) from the group with “neither good nor bad” financial status. No significant differences were detected between the group with the lowest financial status, but this may be due to the underrepresentation of this group (only 2.5% of all respondents). The impact of financial situation confirms the correlations shown in previous studies [19,37] and is certainly inextricably linked to the higher prices of organic products, which is a barrier for many consumers and is discussed later. The relationship between financial status and organic food consumption is particularly confirmed in the group with occasional consumption (*p* = 0.003), which may indicate that, for such consumers, the cost of organic products is more important than for regular eco-con, who, accustomed to certain products, may pay less attention to their price.

In this study, a higher proportion of regular female eco-consumers was recorded in the group of respondents under 35 years old. In relation to the entire study group, and among regular eco-con, it was the younger respondents who had a slightly higher median intake, but these results are not statistically significant. The results obtained by other authors regarding the influence of age on organic food consumption are still inconclusive. Certain authors indicate a higher consumption of organic food among younger women- 25–39 y.o. [36] while others indicate the group is 25–45 y.o. [19]. The Coalition for the BIO Report (2021), on the other hand, shows the most frequent consumption among those aged 36–45 y.o., but also high values among consumers aged 25–35y.o. and 46–55 y.o. [24]. The lack of statistical correlations obtained in the present study, as well as the discrepancies in the studies of other authors, may indicate that the consumption of organic food does not clearly depend on the age of consumers.

Kułyk and Michałowska (2019) showed that the level of organic food consumption significantly depends on the number of people in the household, with the highest level among families of 3–4 people [29]. Studies on organic food e-commerce customers indicate higher consumption in families of five people or more [37]. Studies have shown that consumers tend to be residents of large cities [19,24]. In the present study, higher consumption was noted in residents of rural areas and small towns with up to 10,000 inhabitants and in large families—with five or more people in the household and with more than one child—but these results are not statistically significant. Interestingly, the two factors may be related in Poland according to the Central Statistical Office (in Polish: Główny Urząd Statystyczny—GUS), the fertility rate in less urbanized areas is higher (9.6%) compared to cities (9%), and households have more members [38]. It may be possible that, in the case of respondents from small towns, residence allows for direct access to organic food producers, increased trust in food produced by someone in the neighborhood or the opportunity to grow their own food in line with organic production principles. A study by Żakowska-Biemans and Gutkowska (2018) also showed that, among the consumers who most frequently consumed organic food, rural residents showed the highest proportion. The authors rightly emphasized that the motives for purchasing this type of food are not always altruistic, and that local food or short supply chains (SSCs) meet consumer expectations of freshness of produce and lower price due to lower distribution costs [36]. In addition, mothers in large, rural families may be able to devote more time to caring for food quality due to irregular working hours/no full-time job or a higher concern for the quality of the diet served to numerous household members (children and elderly living in the same households). Different results were obtained by Halicka et al. (2019), where a study involving 2248 inhabitants of rural households in the Mazovian voivodeship showed that, with regard to the choice of baby food, the more children in the household, the greater the importance of convenience of preparation (*p* = 0.003) and the lower the quality of the product confirmed by high-quality symbols (*p* = 0.016). However, this study referred to children’s food choices and was not directly connected with organic food but with sustainable food [39].

Higher consumption was also recorded for families where the mother is raising the child alone (single/divorced/widowed), but this result is most likely distorted by the low number of female respondents (only 7% of respondents). On the other hand, it is worth noting that in such families the income per person is higher so that perhaps the mothers can afford a special diet for their children. 

It is worth pointing out that the results obtained are in line with those of other authors, who are also unable to point out clearly the influence of socio-economic factors. A study conducted by Torjusen et al. (2010) on 63,561 pregnant Norwegian women showed that the frequent use of organic food was associated with lower (<24 y.o.) and higher (>40 y.o.) age groups, lower and higher levels of education, low household income and urban living area [40]. As the authors indicated, the associations between socio-demographic and lifestyle variables and eating organic food reflect complexity and indicate that no quick label like “young and idealistic” or “well educated and wealthy” can be applied to describe women who report frequent intake of organic food during pregnancy. In contrast, in a study by Asvatourian et al. (2018) in a random sample of 3000 people living in the southwest of Scotland, factors such as age, income and level of education were shown to influence the level of organic food consumption, while there was no significant influence of other factors analyzed, such as number of people living in the house, number of children living in the house and locality (urban/rural) [41]. In a French study of 68,946 consumers, higher organic food scores were positively associated with female sex, high occupational status or monthly income per household unit and postsecondary graduate educational level [7].

As Vermeira and Verbeke (2006) have shown, it is not learning about specific socio-demographic segments but learning about factors that appeal to consumer attitudes and beliefs that may be helpful in stimulating more sustainable food consumption [42]. The results obtained in our study show a correlation between the regularity of organic food consumption and the frequency of checking for the presence of a certificate on the packaging (*p* < 0.001) and knowledge of the organic certificate (*p* = 0.004). On the other hand, 15% of female respondents were not able to identify an organic food logo, and about 27% did not check for the presence of a certificate on the packaging (Table 7). In the IMAS study (2017), 67% of respondents were unable to identify the correct logo, and among the biggest barriers besides the high price (64% of indications) were “lack of faith that the food is really organic” (25%) and lack of trust in certifications (15%) [19]. In the current study, it was shown that almost 80% of female respondents agreed with the statement that the use of the organic logo is only possible after obtaining permission from the relevant authorities, and only 4% disagreed, which may indicate an increase in confidence in the certification system compared to the IMAS study. On the other hand, 16% disagreed with the statement that certified products give them confidence that the food has been produced according to organic principles, and as many as 27% had no opinion on the subject. Moreover, for both issues mentioned, groups disagreeing with the above statements had the highest median consumption of organic food. Perhaps this relationship is due to the distortions caused by the low numbers of these groups, but it may also indicate a large lack of consumer knowledge or a high lack of confidence in the organic certification system as well as the previously observed lack of confidence in the actual “greenness” of certified products, even among a certain proportion of regular female consumers. Tobler et al. (2011), in a Swiss study, found that a lack of confidence in whether organic food is truly organic may be caused by the wide variety of organic product packaging labels and the lack of transparency in this area from a consumer perspective [34]. It can therefore be concluded that the awareness of female respondents is quite high in relation to other consumer groups surveyed in Poland, but not yet sufficient. The majority of respondents, on the other hand, are convinced of the important characteristics of organic food and indicate them correctly (Table 8). The largest share of female respondents indicated characteristics such as non-GM/without GMO (81% of respondents), without synthetic additives (68%) and having organic certification (67%). A higher level of awareness is therefore apparent compared to the IMAS (2017) study, where the above characteristics were indicated by 26%, 44% and 16% of respondents, respectively [19]. Thus, it can be concluded that the respondents correctly perceive the important qualities of organic food, but their attitudes and beliefs may indicate an insufficient state of knowledge about organic food and production, so it would be valuable to conduct in-depth analyses of pro-environmental consumer attitudes and to build educational campaigns based on the findings of such studies. 

The results of the survey support hypothesis 3: the most common reason for consuming organic food is health issues, and the most common barrier to consuming organic food is its high price. The results show that, among the study group, health issues account for a total of almost 80% of the indicated reasons for consuming organic food. The reasons cited by most respondents included: it is healthier (69%), contains no artificial additives (58%), is non-GM/without GMO (40%) and contains more nutrients (19%). The results are very similar to those achieved by IMAS (2017) according to which regular consumers buy eco food because it is healthier (69%), does not contain artificial additives or antibiotics (57%), is non-GMO/without GMO (39%) and contains more nutrients (35%) [19]. They are also in line with the results of Bryła (2016), who showed that the most important characteristics of organic food for Polish consumers are healthiness and high quality [43].

It is worth noting that some respondents indicated the impact on the environment among their reasons for purchasing organic food, including concern for the environment (15%) or support for organic farming (12%). These reasons were more important for regular eco-con than for occasional eco-con—20% vs. 12% and 17% vs. 10%, respectively (Figure 1a). A study of Czudec et al. (2022) has revealed stronger environmental motivations. For a significant percentage of organic consumers, the motivation is to support the local economy (51%), of which up to 65% indicated concern for the environment as one of the five most important motives for purchasing organic food [44].

The most frequently cited barrier was price (60% of respondents) and too limited choice of organic products in the shops (48%). As indicated by Bryła’s (2016) research, the critical barriers to the development of the organic food market in Poland were the high price, insufficient consumer awareness, low availability of organic products, short shelf life and low visibility in the shop [43]. The main barriers to organic food consumption, i.e., price and availability, have not changed and are the same as in many studies [26,27,30,45,46,47]. The problem with the availability of organic food has, until recently, particularly concerned organic meat, cold meats, honey and products thereof, fish and seafood and wine [48,49].

The majority of respondents declared that they were able to pay about 20% more for an organic product than for a conventional one, and 13% indicated that they were not able to pay extra at all (Figure 2). This shows an increase compared to the study by Kułyk and Michałowska (2019), where a price that is higher by around 5–10% was accepted, and as many as 38% were not able to pay more for an organic product [45]. A trend was observed that willingness to pay (WTP) a higher price for organic products increases with median consumption, but group 4 declaring a willingness to pay more than 30% of the price does not confirm this trend. This may be as a consequence of being the least numerous (5% of respondents), but it may also be an indication that consumers accept higher prices for organic products up to a certain level (+30%), and above this price difference the purchasing decision becomes a much more difficult issue. This could be an important clue for producers and distributors of eco food.

It is also worth noting that 33% of those surveyed explicitly state among the barriers that they prefer to buy products from a local farmers or market vendors. Unfortunately, in such sources of distribution, one cannot be sure that the organic standards of production have been respected if the product has no information on certification. Respondents may value local/rural products on a level with organic or even consider them superior. Such trends were noted in a study carried out by Żakowska-Biemans and Tekień (2017), which showed that, in the case of eggs, Polish consumers often prefer free-range eggs to organic-certified eggs [50]. There are other studies showing that, for some consumers, geographical proximity (localness) is more important than an organic production system [51,52]. Overall, 30% of respondents also indicate that they prefer home-grown food, which of course may be produced according to organic production rules and have the characteristics of organic production, but until it has undergone a certification process, there is no such certainty and it should not be described as organic.

Summarizing the motivations and barriers to the consumption of organic food, it is worth noting that in the Coalition for the BIO Report (2021), consumers indicated that they would be most driven to increase their purchases of organic food by “lower prices”, the reassurance that “organic food is certainly different from non-organic food” and “production according to ethical standards (e.g., fair trade, good animal husbandry)” and these expectations seem to be holding today [24]. The results of international studies indicate that organic food producers can play a great role in minimizing barriers to purchasing organic food. A study conducted by Ali et al. (2021) found that encouraging Chinese university students to increase the frequency of buying organic food can be achieved by emphasizing fair price, reinforcing confidence in the product, providing health benefits and positive attitudes [53]. In a study by Yu et al. (2021) it was shown that the corporate social responsibility (CSR) image of an organic food company may influence the consumption behavior and co-developing behavior of customers‚—it can effectively promote consumer trust, continuous purchase and active engagement in the co-development of products and services [54].

## 5. Limitations of the Study

When analyzing the presented results, it is important to keep in mind the limitations of the survey. It was conducted in selected towns and selected establishments (purposive controlled selection). On the other hand, respondents entered the survey voluntarily and randomly, so the proportion of women more interested in organic food in our study may have been higher than it would have been in the entire Polish population. Furthermore, only questionnaire sheets indicating the correct definition of organic food were included in the study (59% of all fully completed surveys obtained during the survey). Thus, the surveyed group of women had more knowledge about organic food than the average woman in Poland, which could be related to a higher than average interest in this type of food.

In addition, it should be remembered that the time of the study, the COVID-19 pandemic began in Poland, which, according to numerous studies, had an impact on changing the dietary behavior of Poles [55,56,57], and thus may also have had an impact on the consumption of organic food and its perception among respondents.

## 6. Conclusions

The aim of the study was to assess the frequency of consumption of selected groups of organic products and selected factors influencing it, as well as to analyze the perception of such food by mothers of children under 6 years old living in voivodeships of Eastern Poland. Consumption of organic food in the examined group of Polish mothers of children under 6 years old living in the Mazowieckie, Lubelskie and Podlaskie voivodeships can be described as occasional. However, the study group is dominated by consumption at an average level, which demonstrates a slightly higher consumption than shown in other consumer studies. As the level of education and the financial situation of the household increases, the consumption of organic food is also likely to rise. With regard to the other socio-economic factors, no significant relationships were detected. The most common reason for consuming organic food is invariably health issues, and the most common barrier to consuming organic food is its high price. Organic food, with its associated potential health and environmental benefits, remains inaccessible to people on lower incomes, which contributes to health inequalities in society and should be the subject of policy debate in Poland. A higher level of awareness of the characteristics of organic food and certification is noticeable among respondents compared to other studies but should still be considered as insufficient. It was observed that among regular consumers of organic food, pro-environmental motivations are more important than for occasional consumers, and willingness to pay for organic products increases as the level of consumption of organic food increases as well. It is important to recognize the factors that appeal to consumer attitudes and beliefs in order to carry out effective actions to encourage consumption of organic food. Producers can play a major role in minimizing barriers to buying organic food through appropriate CSR activities that increase credibility and trust in organic food. Further research into organic consumption in Poland and activities to popularize it become particularly important in the context of implementing the concept of sustainable development and the idea of a European Green Deal and should therefore be implemented. Based on the results of the research, recommendations can be made to decision makers and society. First and foremost, regular education on organic food and production is necessary at all levels of education from kindergarten to university. It is also necessary to adequately promote organic food to those who have already completed their education. This applies to all age groups, but especially to young women with small children, as the composition of the food consumed has a major impact on the development of the young organism.

## Figures and Tables

**Table 1:** Indicators for conversion of frequency of consumption of organic products.

Consumption Frequency Categories in the Survey	Daily Frequency (Times/Day)
Several times a day	2
Once a day	1
Several times a week	0.5
Once a week	0.14
1–3 times a month	0.06
Never	0

**Table 2:** Method of interpretation of the results obtained from the question on frequency of consumption of organic products.

Range of Obtained Sum of Frequency of Daily Consumption of Organic Products (Times/Day)	Average Frequency of Consumption of the Organic Products Analyzed Corresponding to the Range of the Sum of Points	Level of Consumption of Organic Products	Organic Food Consumer Category (Acronym)
17.0–34.00	At least once a day	very high	regular ecoconsumer (regular eco-con)
8.5–16.99	Several times a week but not every day	high	
2.38–8.49	At least once a week	medium	
1.02–2.37	Several times a month but less than once a week	low	occasional ecoconsumer (occasional eco-con)
0.01–1.01	At least once a month	very low	
0	Never	no consumption	non-ecoconsumer (non-eco-con)

**Table 3:** Participant characteristics (*n* = 667).

	Lubelskie Voivodeship	Podlaskie Voivodeship	Mazowieckie Voivodeship	Total	% of Total (%)
Number of Respondents	181	175	311	667	100
Age of Respondents					
under 35 years old	82	84	160	326	49
35 years old or more	99	91	151	341	51
Educational Level					
Secondary	27	33	43	103	15
Higher	154	142	268	564	85
Household Financial Situation					
good and very good	157	151	263	571	85
neither good nor bad	21	18	40	79	12
bad and very bad	3	6	8	17	3
Place of Residence					
city >500,000 inhabitants	-	-	165	165	25
city of 100,000 to 500,000 inhabitants	26	36	56	118	18
city of 10,000 to 100,000 inhabitants	97	114	59	270	41
rural areas and cities with up to 10,000 inhabitants	58	25	31	114	17
Number of People in the Household					
2–3	55	39	111	205	31
4	88	86	151	325	49
5 and more	38	50	49	137	21
Number of Young Children (Up to 6 Years Old) in the Household					
1	106	91	189	386	58
2 and more	75	84	122	281	42
Civil Status					
married/partnership	168	167	285	620	93
single/divorced/widowed	13	8	26	47	7

**Table 4:** Self-reported frequency of organic food groups consumption during last three months among Polish mothers (percentage of respondents for each frequency (%), sums up to 100% in the row), *n* = 667.

Nr.	Organic Product Group	Several Times a Day	Once a Day	Several Times a Week	Once a Week	1–3 Times Per Month	Never
1	bread	3.1	6.4	8.7	7.8	30.6	**43.3**
2	white rice, plain pasta, fine groats	0.0	1.3	7.5	10.6	32.1	**48.4**
3	buckwheat groats, oatmeal, whole wheat pasta	0.6	2.8	10.6	9.4	33.3	**43.2**
4	butter	2.7	5.2	4.5	3.3	21.1	**63.1**
5	milk	1.6	5.2	6.0	5.7	23.5	**57.9**
6	fermented dairy beverages, e.g., yogurt and kefir	0.4	4.5	10.2	10.6	28.5	**45.7**
7	cottage cheese, processed and ripened cheese	0.3	1.5	7.0	8.8	30.1	**52.2**
8	semi-hard cheeses	0.1	2.2	6.0	8.1	24.3	**59.2**
9	eggs	3.4	12.1	**40.9**	14.4	14.1	14.8
10	vegetables	7.6	12.7	**27.7**	10.9	23.1	17.8
11	processed vegetables	2.4	5.4	21.1	9.6	26.8	**34.6**
12	fruit	5.2	9.6	22.5	12.0	**28.6**	22.0
13	fruit preserves	1.8	5.4	16.9	11.7	28.5	**35.7**
14	processed meats	1.2	6.3	11.4	12.7	29.5	**38.8**
15	red meat, e.g., pork and beef	0.4	1.6	5.7	10.2	27.0	**55.0**
16	white meat, e.g., chicken, turkey and rabbit	0.3	2.2	10.8	13.5	32.5	**40.6**
17	fish	0.4	0.4	3.0	10.2	23.2	**62.7**
18	legumes, e.g., beans, peas and lentils	0.7	1.0	5.2	6.9	27.9	**58.2**
19	ready meals	0.1	0.3	1.0	3.7	14.2	**80.5**
20	confectionery, biscuits, chocolate bars and sweets	0.4	0.1	4.6	3.9	27.9	**63.0**
21	honey	2.5	6.1	17.1	12.7	30.4	**31.0**
22	coffee	1.6	2.4	2.5	1.8	11.2	**80.4**
23	teas	2.2	2.5	5.8	4.3	11.8	**73.2**
24	alcoholic beverages	0.6	0.1	0.4	0.9	5.7	**92.2**

bold values highlight the consumption frequencies indicated by the highest proportion of female respondents.

**Table 5:** Level of organic food consumption in the surveyed group of respondents (*n* = 667).

Level of Consumption of Organic Products Analyzed	Number of Respondents (*n* = 667)	Share of Respondents (%)	Organic Food Consumer Category	Number of Respondents (*n* = 667)	Share of Respondents (%)
very high	1	0.1	regular eco-con	290	43.5
high	36	5.4			
medium	253	37.9			
low	162	24.3	occasional eco-con	342	51.3
very low	180	27.0			
no consumption	35	5.2	non-eco-con	35	5.2

**Table 6:** Relationship between selected socio-economic factors and level of organic food consumption.

Socio-Economic Elements Examined		Proportion of Respondents Characterized by Particular Socio-Ecological Conditions (% Respondents)					Consumption of Organic Food among Respondents (Multiples/Day) (Mediana/Min-Max)			
		All *n* = 667	Non Eco-Con *n* = 35	Occasional Eco-Con *n* = 342	Regular Eco-Con *n* = 290	Chi-Square *p*-Value	All *n* = 667	Occasional Eco-Con *n* = 342	Regular Eco-Con *n* = 290	Kruskal-Wallis *p*-Value
Age	under 35	48.9	4.3	50.9	44.8	0.504	2.05 0.00–21.55	0.87 0.06–2.37	4.40 2.40–21.55	NS
	35 and over	51.1	6.2	51.6	42.2		1.78 0.00–16.38	1.00 0.03–2.33	4.39 2.41–16.38	
Educational level	Secondary	15.4	9.7	55.3	35.0	**0.041**	1.45 a 0.00–21.55	0.80 0.03–2.25	5.20 2.70–21.55	**0.036 a**
	Higher	84.6	4.4	50.5	45.0		2.02 a 0.00–13.82	1.00 0.06–2.37	4.35 2.40–13.82	
Financial situation	Bad and very bad	2.5	17.6	41.2	41.2	**0.038**	1.89 0.00–8.15	0.90 0.44–1.96	5.53 2.83–8.15	**0.003 a** **0.016 b**
	Neither good nor bad	11.8	6.3	63.3	30.4		0.98 a 0.00–11.69	0.68 b 0.06–2.09	3.93 2.73-11.69	
	Very good and good	85.6	4.7	49.9	45.4		2.03 a 0.00–21.55	1.02 b 0.03–2.37	4.46 2.40–21.55	
Place of residence	Rural areas and cities with up to 10,000 inhabitants	17.1	2.6	49.1	48.2	0.429	2.28 0.00–16.38	1.01 0.03–2.33	4.37 2.41–16.38	NS
	city with 10–100 thousand inhabitants	40.5	4.4	51.9	43.7		1.94 0.00–21.55	1.00 0.06–2.37	4.37 2.40–21.55	
	city with 100–500 thousand inhabitants	17.7	5.9	49.2	44.9		1.89 0.00–12.79	0.66 0.06–2.15	4.06 2.72–12.79	
	city >500 thousand inhabitants	24.7	7.9	53.3	38.8		1.65 0.00–13.81	1.01 0.06–2.34	4.83 2.45–13.81	
Region of residence	Lubelskie	27.1	4.4	53.6	42.0	0.099	1.86 0.00–13.82	0.98 0.03–2.33	4.38 2.40–13.82	NS
	Podlaskie	26.2	4.6	43.4	52.0		2.49 0.00–21.55	0.97 0.06–2.37	4.18 2.42–21.55	
	Mazowieckie	46.6	6.1	54.3	39.6		1.67 0.00–13.81	0.99 0.06–2.34	4.70 2.45–13.81	
People living in the house	2–3	30.7	3.9	56.6	39.5	0.313	1.79 0.00–13.52	1.01 0.09–2.30	5.13 2.40–13.52	NS
	4	48.7	6.5	49.2	44.3		1.97 0.00–21.55	0.85 0.03–2.37	4.30 2.41–21.55	
	5 or more	20.5	4.4	48.2	47.4		2.13 0.00–16.38	1.01 0.06–2.31	4.02 2.41–16.38	
Children under 6 y.o. living in the house	1	57.9	6.2	51.8	42.0	0.337	1.78 0.00–13.81	0.99 0.06–2.33	4.70 2.40–13.81	NS
	2 or more	42.1	3.9	50.5	45.6		2.11 0.00–21.55	0.87 0.03–2.37	3.93 2.43–21.55	
Civil status	married/partnership	93	5.2	51.9	42.9	0.462	1.85 0.00–21.55	0.98 0.03–2.37	4.30 2.40–21.55	NS
	single/divorced/widowed	7	6.4	42.5	51.1		2.42 0.00–13.45	0.83 0.20–2.34	6.01 2.42–13.45	

NS—no statistical significance; statistical significance at *p*-value < 0.05 are bolded; groups with statistically significant relationships denoted by the same letters (a, b).

**Table 7:** Relationship between knowledge of selected consumer issues related to organic food and the level of organic food consumption.

Selected Consumer Issues Related to Organic Food		Share of Respondents Giving Individual Answers (% of Respondents)					Consumption of Organic Food among Respondents (Multiples/Day) (Median/Min-Max)			
		All *n* = 667	Non Eco-Con *n* = 35	Occasional Eco-Con *n* = 342	Regular Eco-Con *n* = 290	Chi-Square *p*-Value	All *n* = 667	Occasional Eco-Con *n* = 342	Regular Eco-Con *n* = 290	Kruskal-Wallis *p*-Value
When buying organic products, do you make sure that there is an official mark of certified organic food on the packaging?	Yes	31.0	0.5	42.5	57.0	**<0.001**	2.78 a 0.00–13.82	1.01 b 0.06–2.32	4.62 c 2.42–13.82	**<0.001 a** **<0.001 b** **0.048 c**
	Sometimes	42.3	0.7	52.1	47.2		2.20 a 0.00–21.55	1.02 b 0.06–2.37	4.51 2.40–21.55	
	No	26.7	18.0	60.1	21.9		0.79 a 0.00–12.79	0.76 b 0.03–2.34	3.48 c 2.41–12.79	
Correctness of the indication of the organic certificate	Yes	84.7	4.2	50.3	45.5	**0.004**	2.03 a 0.00–16.38	1.01 0.06–2.37	4.51 2.40–16.38	**<0.001**
	No	15.3	10.8	56.9	32.4		1.03 a 0.00–21.55	0.83 0.03–2.30	3.77 2.60–21.55	
Only with certified products can I be sure that the food has been produced in accordance with the principles of organic production.	I agree	56.5	5.3	51.2	43.5	0.302	1.85 0.00–21.55	0.92 0.06–2.37	4.56 2.40–21.55	NS
	I disagree	16.5	7.3	43.6	49.1		2.20 0.00–16.38	1.23 0.03–2.21	4.29 2.43–16.38	
	I have no opinion	27.0	3.9	56.1	40.0		1.79 0.00–13.82	0.85 0.06–2.34	4.42 2.41–13.82	
The use of the organic food logo on the packaging is only possible with permission from the relevant authorities.	I agree	78.0	4.8	51.3	43.8	0.162	1.91 0.00–21.55	1.00 0.03–2.37	4.51 2.40–21.55	NS
	I disagree	4.0	0.0	44.4	55.6		2.54 0.12–12.60	0.78 0.12–2.12	5.68 2.43–12.60	
	I have no opinion	18.0	8.3	52.5	39.2		1.52 0.00–16.38	0.73 0.06–2.32	3.94 2.41–16.38	

NS—no statistical significance; statistical significance at *p*-value < 0.05 are bolded; groups with statistically significant relationships denoted by the same letters (a, b, c).

**Table 8:** Characteristics of organic food as perceived by the respondents (multiple choice).

Characteristics of Organic Food	Number of Indications	Percentage of Indications (%) *n* = 2510	Percentage of Respondents (%) *n* = 667
Without GMO/GMO-free	541	21.6	81
Free of synthetic additives	455	18.1	68
Organically certified	445	17.7	67
Natural	322	12.8	48
Healthy food	223	8.9	33
Environmentally friendly	135	5.4	20
Contains beneficial nutrients	108	4.3	16
Fresh/seasonal produce	88	3.5	13
Expensive	54	2.2	8
From the countryside	50	2.0	7
Local	25	1.0	4
Traditional	22	0.9	3
Tasty	18	0.7	3
Helps to cure illnesses	13	0.5	2
Fashionable	11	0.4	2
Dietary/nutritional	0	0.0	0

**Table 9:** Reasons for using organic food by respondents (multiple choice possible).

Reasons for Consuming Organic Food	Number of Indications	Percentage of Indications (%) *n* = 1555	Percentage of Respondents (%) *n* = 667
It is healthier	457	29.4	69
It contains no artificial additives (preservatives, flavors, colors)	384	24.7	58
Is not genetically modified (without GMO)	265	17.0	40
Contains more nutrients (vitamins, minerals, etc.)	127	8.2	19
Is tastier	108	7.0	16
Out of concern for the environment	100	6.4	15
I want to support organic farming	82	5.3	12
Helps to treat illnesses/allergies	28	1.8	4
Is a fashionable food	4	0.2	1

**Table 10:** Barriers to consumption of organic food for respondents (multiple choice).

Barriers to the Consumption of Organic Food	Number of Indications	Percentage of Indications (%) *n* = 1624	Percentage of Espondents (%) *n* = 667
Organic food is too expensive/not worth it	401	24.7	60
There is too little choice of organic products in the shops	320	19.7	48
I prefer to buy products from the local farmer, market or marketplace	217	13.4	33
I prefer home-grown food	198	12.2	30
Organic food shops are too far away	145	8.9	22
I do not know organic food producers	95	5.8	14
Not available/I have not seen it in shops	79	4.8	12
I do not trust certificates on products	74	4.6	11
I have no need for it/it does not matter to me	45	2.8	7
I cannot recognize organic food/do not know how it is labelled	24	1.5	4
I am disappointed with the quality/taste	14	0.9	2
Organic food is no different from conventional food	12	0.7	2

## Supplementary Materials

The following are available online at https://www.mdpi.com/article/10.3390/ijerph192215196/s1. Figure S1: Flow chart of the project.
