# Supplementary figures and images for: Organic Food Consumption and Perception among Polish Mothers of Children under 6 Years Old
Source: Int J Environ Res Public Health. 2022 Nov 17;19(22):15196. doi: 10.3390/ijerph192215196 (PMC9690996; doi:10.3390/ijerph192215196)

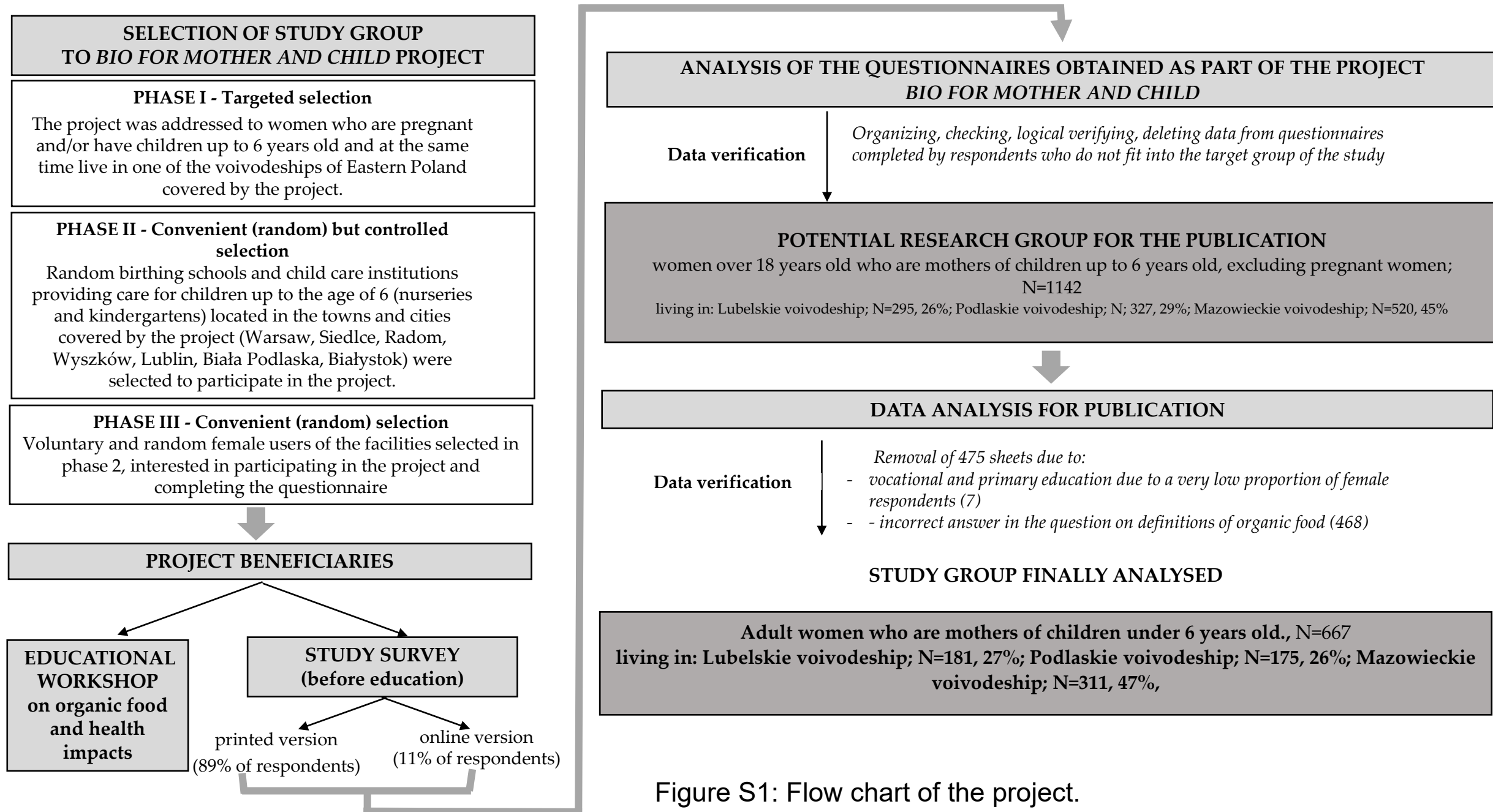

Figure S1: Flow chart of the project.

Supplement: Supplementary file 1 [file ijerph-19-15196-s001.zip › ijerph-2006003-supplementary.pdf]
